# Analysis of Macular Vascularization Using Optical Coherence Tomography Angiography in Patients with Obstructive Sleep Apnea Syndrome: A Prospective Clinical Study

**DOI:** 10.3390/medicina60050757

**Published:** 2024-05-02

**Authors:** Laura Abdessater, Matthieu Hein, Florence Rasquin

**Affiliations:** 1 Hôpital Universitaire de Bruxelles, Service de Médecine Interne, Université Libre de Bruxelles, ULB, 1070 Bruxelles, Belgium; laura.abdessater@hubruxelles.be; 2 Hôpital Universitaire de Bruxelles, Service de Psychiatrie et Laboratoire du Sommeil, Université Libre de Bruxelles, ULB, 1070 Bruxelles, Belgium; 3 Laboratoire de Psychologie Médicale et Addictologie (ULB312), Université Libre de Bruxelles, ULB, 1020 Bruxelles, Belgium; 4 Hôpital Universitaire de Bruxelles, Service d’Ophtalmologie, Université Libre de Bruxelles, ULB, 1070 Bruxelles, Belgium; florence.rasquin@hubruxelles.be

**Keywords:** obstructive sleep apnea syndrome, vascular density, retinal vascular network, superficial capillary plexus, deep capillary plexus, optical coherence tomography angiography

## Abstract

*Background and Objectives:* Given the conflicting data available in the literature, this study aimed to investigate the impact of obstructive sleep apnea syndrome (OSAS) on the macular vascular density (VD) and perfusion density (PD). *Materials and Methods:* Based on the obstructive apnea–hypopnea index (OAHI), 61 prospectively recruited patients were assigned to either a control group (*n* = 12; OAHI < 5/h) or an OSAS group (*n* = 49; OAHI ≥ 5/h). The macular VD and PD of the superficial and deep capillary plexuses (SCP and DCP, respectively) were measured in the parafoveolar and perifoveolar areas using Zeiss PLEX Elite 9000 (6 × 6 mm). The values were compared between the control and OSAS groups. *Results:* Compared with the control group, the OSAS group demonstrated an increased VD of the DCP in the parafoveolar and perifoveolar areas and PD of the DCP in the perifoveolar area. No significant differences in either the macular VD or PD of the SCP were observed. There was no correlation between the OAHI and macular VD or PD. *Conclusions:* This study indicates that collateral vessel formation and possible retinal vasodilation occur in the DCP of patients with OSAS.

## 1. Introduction

Obstructive sleep apnea syndrome (OSAS) is a major public health issue. The prevalence of OSAS in the global population is difficult to assess, as it is underdiagnosed. OSAS is associated with an increased risk of cardiovascular events and diabetes. If left untreated, it can result in difficult-to-control high blood pressure, heart rhythm disorders, and blood hypercoagulability [1]. OSAS is also associated with heart failure, pulmonary hypertension, and chronic kidney disease [1]. Risk factors for OSAS include obesity, age, male sex, African ethnicity, smoking, and certain craniofacial morphologies [2].

OSAS is characterized by repeated episodes of complete (apnea) or partial (hypopnea) obstruction of the upper airway during sleep, resulting in episodes of oxygen desaturation, hypercapnia, and micro-arousals that impair sleep quality [2,3]. It is diagnosed when polysomnography reveals an obstructive apnea–hypopnea index (OAHI) greater than 5 (i.e., at least five episodes of obstructive apnea/hypopnea per hour of sleep). Intermittent hypoxia induces hyper-activation of the sympathetic nervous system, inflammatory response, and oxidative stress. These are the primary mechanisms responsible for the cardiovascular and metabolic consequences associated with OSAS [4]. Acute hypoxia also induces self-regulated retinal vasodilation [5].

Studies have suggested that OSAS is associated with various ophthalmologic conditions, such as non-arteritic anterior ischemic optic neuropathy, retinal vein occlusion, glaucoma, central serous chorioretinopathy, and severe diabetic macular edema [6,7]. Several studies have assessed choroidal thickness in patients with OSAS; however, these studies have presented divergent results. One meta-analysis concluded that there was a significant decrease in choroidal thickness in patients with OSAS [8].

Optical coherence tomography angiography (OCTA) is an imaging technique that allows visualization of the retinal and choroidal vasculatures without contrast injection. The retina is irrigated by two vascular networks: retinal and choroidal vascularization. OCTA highlights the superficial and deep capillary plexuses (SCP and DCP, respectively) of the retinal vascular network (RVN). Limited studies have investigated the RVN density using OCTA in patients with OSAS, and the available evidence is inconsistent. For example, Yu et al., who were among the first to investigate the macular perfusion density (PD) in patients with OSAS, reported a decrease in the density in the parafoveolar area with increasing OSAS severity [9]. Meanwhile, Moyal et al. reported no significant differences in the macular PD between their control, mild OSAS, moderate OSAS, and severe OSAS groups [10]. Additionally, while Cai et al. observed a significant increase in the PD in the parafoveolar and perifoveolar DCP regions in patients with severe OSAS compared with that in controls, no difference was seen in the PD of the SCP [3].

The Ophthalmology Department of Erasme Hospital in Brussels is equipped with PLEX Elite 9000. Owing to its powerful algorithm, this equipment allows accurate quantitative analysis of the retinal vascular density in terms of both the PD and total length of perfused vessels (VD). This study primarily assesses the retinal vascular density and has the following objectives:

(1) To assess the macular VD of both the SCP and DCP in patients with OSAS;
(2) To assess the macular PD of both the SCP and DCP in patients with OSAS;
(3) To assess the possible correlation between the macular VD and OSAS severity.

## 2. Materials and Methods

### 2.1. Methodology

Patients were recruited from the Sleep Laboratory of Erasme Hospital (SLEH). Polysomnography was previously indicated by each patient’s physician. The study was verbally described to all patients scheduled for polysomnography either on the day of the procedure or the following day. Written informed consent was obtained from all patients included in the study. Patient recruitment, data collection, ophthalmologic examination, and ocular imaging were performed by the same investigator during each patient’s hospitalization.

#### 2.1.1. Study Design and Population

This single-center prospective observational clinical study assessed SLEH patients between January and March 2021. The inclusion criteria for this study were to sign informed consent for participation and to stay at the SLEH between January and March 2021 to benefit from a polysomnographic examination. The exclusion criteria for this study were OSAS already treated by CPAP; ongoing oxygen therapy; history of retinal surgery, retinopathy and/or maculopathy, ocular trauma, or glaucoma; best-corrected visual acuity (BCVA) of ≤7/10 on the Monoyer scale (≥0.2 in LogMar) or a spherical equivalent (SE) of <−6 diopters (strong myopes) in both eyes. A control group and a study group were formed on the basis of the OAHI following the results of polysomnography. Patients with an OAHI of ≥5 and <5 were assigned to the OSAS group and the control group, respectively. Ophthalmologic examinations were performed blind (i.e., the investigator did not know whether a patient had OSAS or not). 

#### 2.1.2. Data Collection

Age, sex, body mass index, smoking status, and medical history were collected from all patients included in this study by a physician during the routine admission interview based on a standardized form specific to the SLHE. The patients underwent an ophthalmologic clinical examination, including refractometry, forced-air tonometry, and BCVA assessment using the Monoyer scale. Each patient then underwent ocular imaging using a wide-angle fundus imaging software (Zeiss Clarus 500), optical coherence tomography (OCT, Heidelberg Engineering Spectralis), Enhanced Depth Imaging OCT (OCT-EDI) of the macular region, and OCTA (Zeiss PLEX Elite 9000, version 1.7, Swept-Source OCTA). Refraction (SE), BCVA, intraocular pressure (IOP) by pulsed air, macular thickness, choroidal thickness in the foveolar area, and macular VD and PD were measured in both eyes of each patient. The polysomnography results were reported by the physician in charge of the SLEH 3 weeks after the examination was performed. The data were collected and managed via Erasme Hospital’s secure electronic platform, REDCap.

#### 2.1.3. Polysomnography

Polysomnographic examinations were performed at the SLEH in accordance with the recommendations of the AASM [11]. The polysomnographic setup used is described in Annex S1. Polysomnographic recordings were visually scored by specialized technicians in accordance with the AASM criteria [12]. Annex S1 shows the criteria for obstructive sleep apnea and hypopnea and the calculation of the OAHI. OSAS was considered absent when the OAHI was <5/h and present when the OAHI was ≥5/h. The OSAS group included mild (OAHI of 5–14), moderate (OAHI of 15–29), and severe (OAHI of ≥30) OSAS cases [13].

#### 2.1.4. Ocular Imaging Data Processing

##### OCT and OCT-EDI

Macular and choroidal thicknesses were measured at the foveola by an experienced investigator.

##### OCTA

OCTA enables the three-dimensional visualization of retinal and choroidal vascular structures without contrast injection or contact. It is based on the principle of detecting the movements of diffracting particles, such as erythrocytes. Each B-scan is repeated several consecutive times at the same retinal location to detect moving pixels (i.e., erythrocytes) and stationary pixels, thus revealing blood vessel flow [14], to generate the retinal microvascular structure images.

PLEX Elite 9000 features an interferometric technique that provides three-dimensional structural information using backscattered infrared light at a wavelength of 1060 nm and a speed of 100,000 A-scan/s [15]. The axial resolution is 1.95 µm, and the lateral resolution is 20 µm [16].

**Figure 1:**
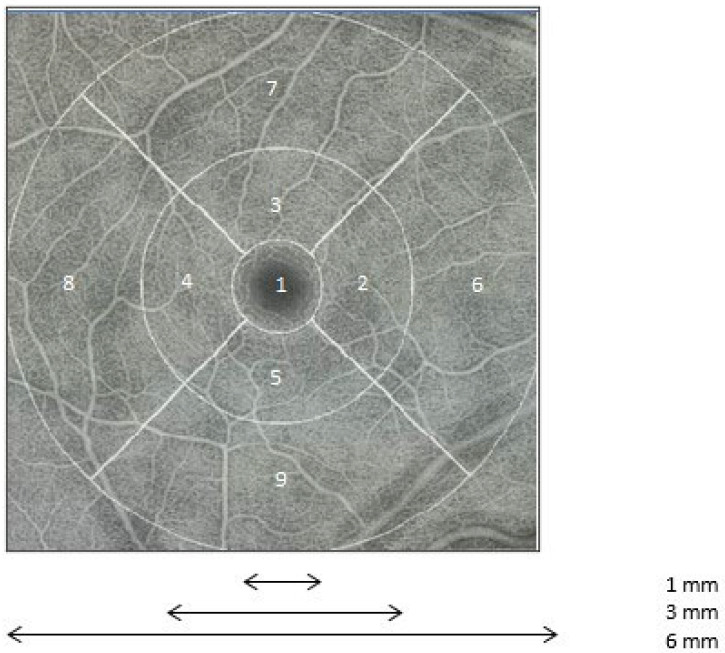
Segmentation of the macula according to the Early Treatment Diabetic Retinopathy Study grid. 1: center; 2: nasal (3 mm); 3: superior (3 mm); 4: temporal (3 mm); 5: inferior (3 mm); 6: nasal (6 mm); 7: superior (6 mm); 8: temporal (6 mm); 9: inferior (6 mm); 1–5: disk (3 mm); 2–5: parafoveolar ring; 6–9: perifoveolar ring; 1–9: disk (6 mm).

Calibrated 6 × 6 mm retinal captures centered on the fovea were taken. The quality of the images was evaluated on the basis of two scores automatically generated by the device and of the visual quality control of each image performed by the investigator (Annex S2). The data were then exported to the Advanced Retina Imaging Network platform. The macular VD in the nine areas of the macula were calculated using the latest version of the Zeiss algorithm (version 0.7.3), as described in the Early Treatment Diabetic Retinopathy Study (ETDRS grid, Figure 1) [15]. The PD in the parafoveolar and perifoveolar areas was also calculated. All measurements were calculated in the SCP, DCP, and RVN. The anatomical location of the capillary plexuses is described in Annex S3.

### 2.2. Statistical Analysis

Only one eye of each patient was selected for the statistical analysis. The eye with the best OCTA image quality was selected when the image quality of both eyes was not identical. When the image quality of the two eyes was identical, the choice was made randomly. The normality of distribution of the data was checked using histograms, scatter boxes, and quantile–quantile plots. Equality of variances was verified using Levene’s test. Categorical variables were described using percentages and numbers, while continuous variables were described according to their mean (±standard deviation) or median distribution (interquartile range, p25–p75). Normally distributed data were analyzed using unpaired Student’s *t*-tests and skewed or dichotomous data using Wilcoxon and chi^2^ tests. Spearman correlation tests were used for correlation analyses. Results were considered significant when the *p*-value was <0.05. Statistical analyses were performed using Stata (version 14).

## 3. Results

### 3.1. Sample Characteristics

The sample comprised 61 patients divided into two groups according to their OAHI. The patients without OSAS (OAHI < 5) were assigned to the control group (*n* = 12) and those with OSAS (OAHI > 5) to the study group (*n* = 49). The numbers of mild (*n* = 17), moderate (*n* = 17), and severe (*n* = 15) OSAS cases were similar. The OSAS and control groups were compared, and the results are described in Table 1. There were no significant differences between the groups in terms of age, sex, or cardiovascular risk factors. The polysomnographic and ophthalmologic variables are described in the second part of Table 1. The sleep period time and sleep efficiency significantly decreased in the OSAS group. The microarousal index and desaturation index were significantly higher, and the time below 90% oxygen saturation was significantly longer in the OSAS group than in the control group. There were no significant differences in the BCVA, SE, macular thickness, or choroidal thickness between the groups. An increase in the IOP was observed in the OSAS group. No ophthalmologic pathologies were observed during wide-angle fundus and OCT examinations.

### 3.2. VD and PD

The OSAS group demonstrated a significantly increased VD in the parafoveolar and perifoveolar DCP and PD in the perifoveolar DCP. No significant differences in the VD and PD of the SCP or RVN were seen (Table 2, Table 3, Table 4 and Table 5) (Annex S4).

### 3.3. Correlation between the VD and OSAS Severity

There was no correlation between the VD and OAHI among the patients with OSAS (Table 6).

## 4. Discussion

This study compared the VD and PD between patients with OSAS and control patients. There were significant increases in the VD and PD of the DCP in the perifoveolar area in the OSAS group compared with those in the control group. The VD of the DCP in the parafoveolar area also significantly increased in the OSAS group. There were no significant differences in the VD and PD of the SCP and RVN. To our knowledge, this study is the first to analyze the macular vascular density in terms of vessel length in patients with OSAS. Nevertheless, several studies have previously analyzed the PD in these patients.

The current results are consistent with those of Cai et al., who reported an increase in the PD in the parafoveolar and perifoveolar DCP in the severe OSAS group compared with that in the control group [3]. Furthermore, Moyal et al., Cai et al., and Colak et al. all reported no significant difference in the PD of the SCP between groups [3,10,17]. This is because the SCP consists of capillaries, arterioles, and venules, whereas the DCP consists of only capillaries and venules [16,18]. Therefore, the oxygen supply is more stable in the SCP owing to the direct connection to the retinal arterioles from the central retinal artery. This could explain why hypoxia affects the DCP first [17].

The current results demonstrate some discrepancy with previous reports regarding the alteration of the PD of the DCP. Colak et al. and Ucak et al. observed a significant decrease in the PD of the DCP in the parafoveolar area in their OSAS groups [17,19]. Colak et al. also observed this phenomenon in the perifoveolar area [17]. Similarly, Yu et al. reported a significant decrease in the PD of the RVN in the parafoveolar and perifoveolar regions [9]. This discrepancy in results could be explained by differences in ethnicity, age, and duration of OSAS symptoms between the study populations. For example, the populations in the current study and in the study by Cai et al. were younger than the populations studied by Ucak et al. and Colak et al. Furthermore, differences in the definition of the groups, particularly the control group, could reduce the relevance of comparisons between these studies. Moreover, most previous studies used SD RTVue-XR Avanti, which is a less powerful device in terms of image acquisition and analysis than SS PLEX Elite 9000.

The OAHI is the classic index used to evaluate OSAS severity. The current study revealed no correlation between the VD and OAHI in the patients with OSAS. Similarly, Cai et al. and Colak et al. did not report correlations between the PD and OAHI [3,17]. However, Yu et al. and Ucak et al. reported negative correlations between the OAHI and PD [9,19]. Some experts have questioned the definition of OSAS based only on the OAHI, as the OAHI may not be the most reliable indicator of OSAS severity. A new definition of OSAS could include the oxygen desaturation index (ODI) [20]. Cai et al. supported the inclusion of the oxygen saturation in the definition of OSAS, as their results showed a negative correlation between the perifoveolar PD and the lowest hemoglobin oxygen saturation [3]. However, Colak et al. did not report a correlation between the PD of the SCP and DCP and time spent under 90% oxygen saturation [17].

The pathophysiological mechanisms driving alterations in the VD and PD of patients with OSAS are not yet well-understood. It is well-established that OSAS-related intermittent hypoxia results in the activation of the orthosympathetic system, which leads to peripheral vasoconstriction [4,21]. However, unlike choroidal vessels, retinal vessels lack autonomic innervation [5]. Hypoxia and hypercapnia result in retinal vasodilation via an autoregulatory mechanism that maintains appropriate blood flow based on metabolic tissue needs. This autoregulatory mechanism is mediated by local factors, such as vasoactive molecules released by the endothelium. Nitric oxide (NO), certain prostaglandins (i.e., PGI2 and PGE2), and extracellular lactate are involved in retinal arterial vasodilation in response to hypoxia and hypercapnia [4,22]. These physiological mechanisms could explain the increase in the PD of the DCP in the perifoveolar area observed in the patients with OSAS in the current study and in the severe OSAS group in the study by Cai et al. [3].

It is speculated that an increase in the VD in terms of vessel length occurs secondary to hypoxia; however, the exact nature of the vessels (i.e., whether they are neovessels or collateral vessels) is unknown. The non-anarchic organization of the vessels observed in OCTA images supports the hypothesis that they are collateral vessels that develop in response to hypoxia. This phenomenon has been established at the coronary level in patients with chronic ischemic heart disease [23]. One study has suggested that coronary collateral vessels also develop in patients with OSAS [24]. Patients with OSAS have higher blood levels of vascular endothelial growth factor (VEGF) than individuals without OSAS. Intermittent hypoxia stimulates VEGF gene transcription via hypoxia-induced factors [25]. Oxidative stress may also be involved in the development of collateral vessels [26]. The increased levels of VEGF and oxidative stress present in patients with OSAS are likely to be the two mechanisms involved in the collateral vessel formation observed in the current study.

The decrease in the retinal PD described in previous studies could be explained by endothelial dysfunction and atherosclerosis, which may be associated with long-term OSAS [4,27]. According to a recent meta-analysis, severe OSAS is associated with a high risk of endothelial dysfunction [27]. Long-term endothelial dysfunction and oxidative stress could induce a decrease in NO production, which would decrease vasodilation and, therefore, macular vascular perfusion in patients with OSAS [28,29]. The impairment of vascular reactivity, regardless of the relation to endothelial dysfunction, would be more marked in patients experiencing apnea with an oxygen desaturation index of >20 [30]. In the long term, these mechanisms could be responsible for capillary occlusion or destruction at the origin of a PD-related decrease. Therefore, it would be interesting to continue the current study to investigate the VD and PD in the included patients for several years to observe whether a two-step reaction in the retinal vasculature exists in patients with OSAS.

### Limitations

Although this study was a prospective controlled study, it involved a single center, and its power is limited by the number of patients included. In addition, the control group included only subjects with complaints related to sleep but without sleep disordered breathing. Therefore, this group may potentially not be representative of the population without apnea. However, in order to avoid as much as possible any risk of selection bias during recruitment for this study, all subjects eligible according to the inclusion and exclusion criteria were invited to participate. Nevertheless, despite this systematic invitation, only patients who agreed to participate in this study were included, which may potentially limit the generalizability of our results. Furthermore, it was difficult to estimate how long the included patients had experienced OSAS symptoms. Studies with longer follow-up periods are necessary to confirm the pathophysiological hypotheses and clinical implications of the current results.

## 5. Conclusions

This study revealed an increase in the VD of the DCP in the parafoveolar and perifoveolar areas and PD of the DCP in the perifoveolar area in patients with newly diagnosed OSAS, which suggests that collateral vessel formation and possible retinal vasodilation occur in the DCP for this particular subpopulation. Moreover, despite its limitations, this study therefore seems to open new perspectives for better understanding the pathophysiology of ophthalmological complications associated with OSAS. In addition, the identification of these alterations in macular VD and PD related to OSAS could allow the future development of new strategies for the prevention and treatment of ophthalmological complications in patients with OSAS. Finally, studies with long-term follow-up seem to be necessary to explore and confirm the hypothesis of a two-phase pathophysiological mechanism that could explain our results and those of previous studies on older patients with OSAS.

## Figures and Tables

**Table 1:** Description and comparison of study groups.

	Control (*n* = 12)	OSAS (*n* = 49)	*p*-Value
Demographic variables			
Age (years)	36.2 ± 11.4	43.8 ± 13.3	0.071
Sex (M)	50.0%	67.4%	0.262
Body mass index (kg/m^2^)	28.7 ± 8.0	31.9 ± 7.2	0.172
Smoking	16.7%	24.5%	0.564
Diabetes type 2	0.0%	8.2%	0.306
High blood pressure	8.3%	22.5%	0.270
Hypercholesterolemia	16.7%	16.3%	0.977
Chronic obstructive pulmonary disease	0%	0%	
Polysomnographic variables			
Sleep latency (min)	64.5 (39.8–90.5)	58.0 (27.0–96.0)	0.935
Sleep period time (min)	472.0 ± 73.8	406.6 ± 83.3	0.016
Total sleep time (min)	433.2 ± 63.7	384.8 ± 78.0	0.051
Sleep efficiency (%)	80.2 (76.2–80.9)	70.6 (61.9–79.0)	0.014
Stage 1 (%)	7.3 (5.1–8.2)	8.2 (6.2–10.0)	0.179
Stage 2 (%)	46.3 ± 7.7	51.7 ± 10.3	0.095
Stage 3 (%)	20.5 ± 9.9	10.1 ± 8.6	0.001
REM sleep (%)	18.4 ± 7.1	13.6 ± 6.5	0.028
Arousal (%)	7.6 (4.5–11.5)	11.7 (7.5–22.0)	0.022
Number of arousals	24.1 ± 11.9	28.1 ± 13.3	0.347
Micro arousal index	5.0 (5.0–11.5)	15.0 (9.0–23.0)	0.002
Desaturation index	3.5 (2.5–8.0)	14.0 (8.0–37.0)	<0.001
Time under 90% oxygen saturation (min)	0.0 (0.0–0.0)	9.5 (1.5–46.0)	<0.001
Ophthalmologic variables			
Visual acuity (LogMar)	−0.5 (−1.1–0.38)	−0.3 (−1.0–0.38)	0.548
Spherical equivalent	−0.5 (−1.1–0.38)	−0.3 (−1.0–0.38)	0.548
Macular thickness (µm)	228.2 ± 15.2	227.2 ± 20.8	0.876
Choroidal thickness (µm)	334.1 ± 65.3	315.6 ± 81.7	0.469
Intraocular pressure (mmHg)	13.0 ± 2.7	15.3 ± 3.4	0.039

**Table 2:** Vascular density (mm^−1^) of the superficial capillary plexus.

	Control (*n* = 12)	OSAS (*n* = 49)	*p*-Value
SCP DV mean	19.8 ± 1.1	20.4 ± 0.7	0.339
SCP DV central	11.9 ± 2.8	12.7 ± 2.7	0.330
SCP DV nasal (3 mm)	20.2 ± 0.9	20.4 ± 1.3	0.586
SCP DV superior (3 mm)	20.3 ± 1.2	20.4 ± 1.0	0.688
SCP DV temporal (3 mm)	20.1 ± 1.1	20.4 ± 0.9	0.412
SCP DV inferior (3 mm)	20.2 ± 1.1	20.3 ± 1.1	0.792
SCP DV nasal (6 mm)	21.8 ± 0.9	22.0 ± 0.7	0.286
SCP DV superior (6 mm)	20.1 (18.6–20.7)	20.4 (19.8–21.0)	0.284
SCP DV temporal (6 mm)	19.0 (17.1–20.0)	19.1 (18.2–19.5)	0.828
SCP DV inferior (6 mm)	19.7 (19.1–21.0)	20.5 (20.0–20.9)	0.217
SCP DV parafoveolar ring	20.2 ± 1.0	20.4 ± 0.9	0.553
SCP DV perifoveolar ring	20.1 (19.2–20.8)	20.6 (20.1–20.8)	0.211
SCP DV disk (3 mm)	19.3 ± 1.1	19.5 ± 1.0	0.431
SCP DV disk (6 mm)	19.8 ± 1.1	20.2 ± 0.7	0.192

**Table 3:** Vascular density (mm^−1^) of the deep capillary plexus.

	Control (*n* = 12)	OSAS (*n* = 49)	*p*-Value
DCP DV mean	13.1 ± 3.5	15.2 ± 2.7	0.027
DCP DV central	0.8 ± 1.1	1.3 ± 1.5	0.213
DCP DV nasal (3 mm)	13.2 ± 4.3	15.2 ± 2.9	0.064
DCP DV superior (3 mm)	13.2 (11.0–18.2)	17.0 (14.5–17.7)	0.157
DCP DV temporal (3 mm)	12.3 ± 3.6	14.2 ± 3.1	0.067
DCP DV inferior (3 mm)	14.0 ± 4.3	15.6 ± 2.9	0.128
DCP DV nasal (6 mm)	14.2 ± 3.8	16.6 ± 2.7	0.012
DCP DV superior (6 mm)	13.0 ± 3.7	15.1 ± 3.2	0.051
DCP DV temporal (6 mm)	13.6 ± 3.7	15.5 ± 3.1	0.068
DCP DV inferior (6 mm)	13.5 ± 4.3	15.8 ± 3.1	0.033
DCP DV parafoveolar ring	13.4 ± 3.9	15.3 ± 2.7	0.049
DCP DV perifoveolar ring	13.6 ± 3.7	15.8 ± 2.9	0.028
DCP DV disk (3 mm)	12.0 ± 3.6	13.7 ± 2.4	0.047
DCP DV disk (6 mm)	13.2 ± 3.6	15.2 ± 2.7	0.029

**Table 4:** Vascular density (mm^−1^) of the total retinal vascular network.

	Control (*n* = 12)	OSAS (*n* = 49)	*p*-Value
RVN DV mean	20.7 ± 0.9	21.1 ± 0.6	0.074
RVN DV central	12.2 ± 2.7	13.0 ± 2.7	0.374
RVN DV nasal (3 mm)	21.3 ± 1.0	21.5 ± 1.0	0.598
RVN DV superior (3 mm)	21.0 ± 1.2	21.5 ± 0.9	0.097
RVN DV temporal (3 mm)	21.3 ± 1.0	21.7 ± 0.7	0.133
RVN DV inferior (3 mm)	21.3 ± 1.0	21.5 ± 1.0	0.547
RVN DV nasal (6 mm)	22.0 ± 0.8	22.3 ± 0.6	0.152
RVN DV superior (6 mm)	21.0 (20.0–21.6)	21.4 (20.8–21.7)	0.204
RVN DV temporal (6 mm)	20.7 ± 1.3	20.9 ± 1.0	0.456
RVN DV inferior (6 mm)	20.2 (19.8–21.7)	21.6 (21.1–21.8)	0.067
RVN DV parafoveolar ring	21.2 ± 0.9	21.5 ± 0.7	0.208
RVN DV perifoveolar ring	20.8 (20.5–21.8)	21.6 (21.2–21.9)	0.119
RVN DV disk (3 mm)	20.2 ± 1.0	20.6 ± 0.8	0.184
RVN DV disk (6 mm)	20.7 (20.2–21.6)	21.4 (21.0–21.7)	0.099

**Table 5:** Perfusion density (%).

	Control (*n* = 12)	OSAS (*n* = 49)	*p*-Value
SCP DP parafoveolar ring	0.43 ± 0.02	0.44 ± 0.02	0.321
SCP DP perifoveolar ring	0.45 (0.42–0.46)	0.46 (0.44–0.46)	0.276
DCP DP parafoveolar ring	0.27 ± 0.8	0.31 ± 0.6	0.056
DCP DP perifoveolar ring	0.27 ± 0.8	0.32 ± 0.6	0.032
RVN DP parafoveolar ring	0.45 ± 0.2	0.46 ± 0.2	0.121
RVN DP perifoveolar ring	0.46 (0.45–0.48)	0.47 (0.47–0.48)	0.147

**Table 6:** Correlation between the vascular density and OAHI.

	OAHI
SCP DV mean	−0.1833
SCP DV central	0.0535
SCP DV nasal (3 mm)	−0.2493
SCP DV superior (3 mm)	−0.1092
SCP DV temporal (3 mm)	−0.0773
SCP DV inferior (3 mm)	−0.2990
SCP DV nasal (6 mm)	−0.1198
SCP DV superior (6 mm)	−0.0088
SCP DV temporal (6 mm)	−0.1960
SCP DV inferior (6 mm)	−0.2350
SCP DV parafoveolar ring	−0.1791
SCP DV perifoveolar ring	−0.1501
SCP DV disk (3 mm)	−0.1662
SCP DV disk (6 mm)	−0.1428
SCP DV mean	−0.1329
SCP DV central	−0.0467
DCP DV nasal (3 mm)	−0.1342
DCP DV superior (3 mm)	−0.1596
DCP DV temporal (3 mm)	−0.1594
DCP DV inferior (3 mm)	−0.2150
DCP DV nasal (6 mm)	−0.0511
DCP DV superior (6 mm)	−0.1332
DCP DV temporal (6 mm)	−0.1551
DCP DV inferior (6 mm)	−0.1500
DCP DV parafoveolar ring	−0.1835
DCP DV perifoveolar ring	−0.1450
DCP DV disk (3 mm)	−0.1812
DCP DV disk (6 mm)	−0.1522
RVN DV mean	−0.0987
RVN DV central	0.0605
RVN DV nasal (3 mm)	−0.2613
RVN DV superior (3 mm)	0.0189
RVN DV temporal (3 mm)	−0.1071
RVN DV inferior (3 mm)	−0.2943
RVN DV nasal (6 mm)	0.0507
RVN DV superior (6 mm)	0.0015
RVN DV temporal (6 mm)	−0.1303
RVN DV inferior (6 mm)	−0.1079
RVN DV parafoveolar ring	−0.2096
RVN DV perifoveolar ring	−0.0570
RVN DV disk (3 mm)	−0.1189
RVN DV disk (6 mm)	−0.0953

## Data Availability

The data presented in this study are available on reasonable request from the corresponding author.

## Supplementary Materials

The following supporting information can be downloaded at: https://www.mdpi.com/article/10.3390/medicina60050757/s1, Annex S1: Polysomnography: setup and definitions; Annex S2: Quality control of PLEX Elite 9000 OCTA images; Annex S3: Anatomical location of the capillary plexuses; Annex S4: Graphical representations of significant results for macular vascular density and perfusion density.
