# Supplementary material for: Analysis of Macular Vascularization Using Optical Coherence Tomography Angiography in Patients with Obstructive Sleep Apnea Syndrome: A Prospective Clinical Study
Source: Medicina (Kaunas). 2024 May 2;60(5):757. doi: 10.3390/medicina60050757 (PMC11122929; doi:10.3390/medicina60050757)
Supplement: Supplementary file 1 [file medicina-60-00757-s001.zip › medicina-2978032-supplementary.pdf]

Supplementary Data

Annex S1 – Polysomnography: setup and definitions

Assembly of the applied polysomnography

- 2 electrooculogram channels
- 3 electroencephalogram channels (Fz-Ax, Cz-Ax, and Oz-Ax, wherein Ax is a mastoid reference)
- 1 submental electromyogram channel
- 1 electrocardiogram
- Thermistors to detect oronasal airflow
- 1 pulse oximeter
- 1 microphone to record respiratory sounds and snoring
- Several piezoelectric sensors to measure thoracic and abdominal breathing
- Several electrodes to measure leg movements

Definition of obstructive apnea and obstructive hypopnea and calculation of the obstructive apnea–hypopnea index (OAHI)

- Obstructive sleep apnea was defined as a ≥90% decrease in airflow for at least 10 s.
- Obstructive sleep hypopnea was defined as a ≥30% decrease in airflow for at least 10 s, in combination with a 3% decrease in oxygen saturation or followed by microarousal.
- The OAHI was calculated as the total number of obstructive sleep apneas and hypopneas divided by the sleep period in hours.

Annex S2 – Quality control of PLEX Elite 9000 OCTA images

Image quality was evaluated on the basis of two scores automatically generated by the device following image acquisition:

- A score indicating the power of the structure image
- A score indicating the power of the fundus image

Only images with a quality greater than or equal to 8/10 were retained. In addition to this automatic control, a visual quality control was performed by the investigator for each image to verify several elements: clarity of focus, uniform illumination of the fundus image without dark corners, good centering of the ETDRS grid on the fovea, little or no shadow indicating an artifact, and little or no saccade on the “En Face” image. Any image with an issue in at least one of these features was excluded.

Annex S3 – Anatomical location of the capillary plexuses in the macula

The retina is irrigated by two vascular networks: the retinal vascular network (RVN) and the choroidal vascular network. The RVN irrigates the retina on the surface. It consists of four capillary plexuses grouped into two overarching plexuses: the superficial capillary plexus (SCP) and the deep capillary plexus (DCP). The outer retina and the avascular foveolar zone are irrigated by choroidal vascularization.

In normal ocular anatomy, the SCP is located at the ganglion cell layer 40 µm below the inner limiting membrane, while the DCP is located at the inner nuclear layer 130 µm below the inner limiting membrane. The total RVN is located between the inner limiting membrane and Bruch’s membrane, minus 41 µm.

Annex S4 – Graphical representations of significant results for macular vascular density and perfusion density

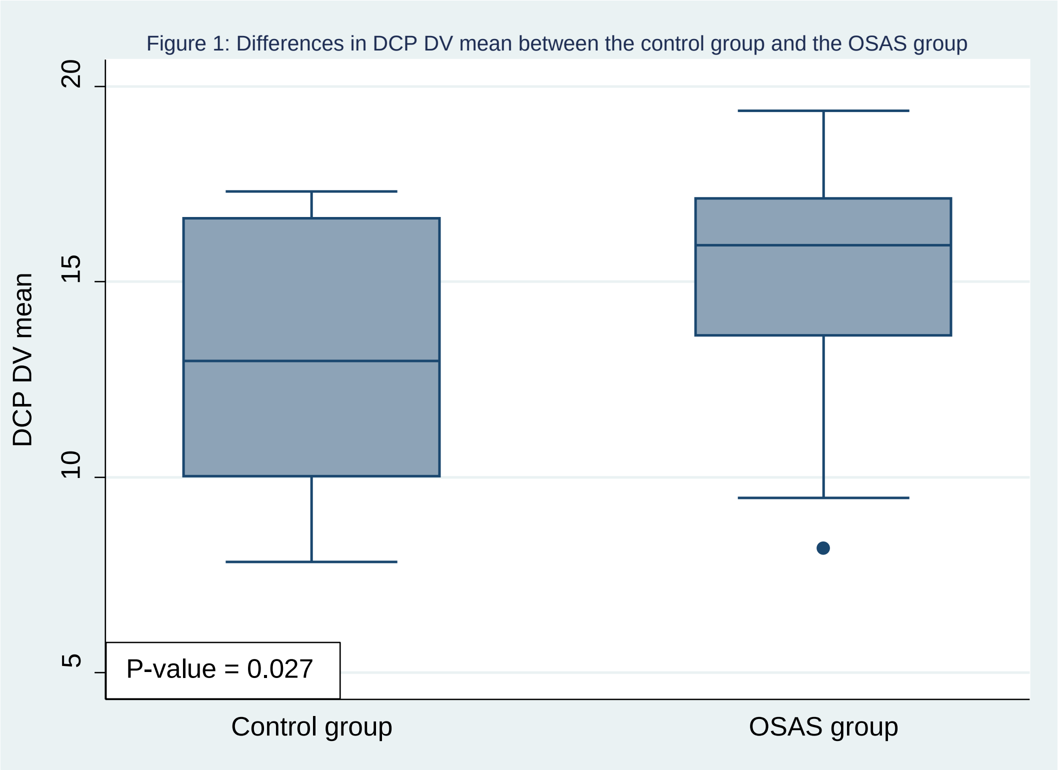

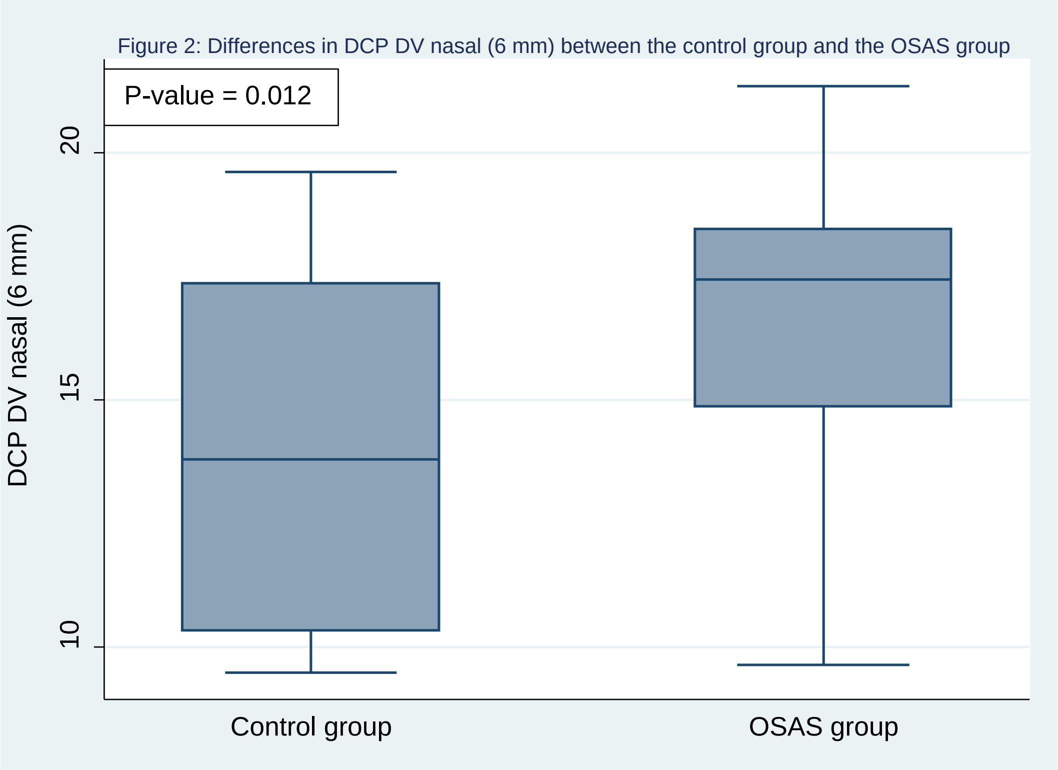

Figure 3: Differences in DCP DV inferior (6 mm) between the control group and the OSAS group

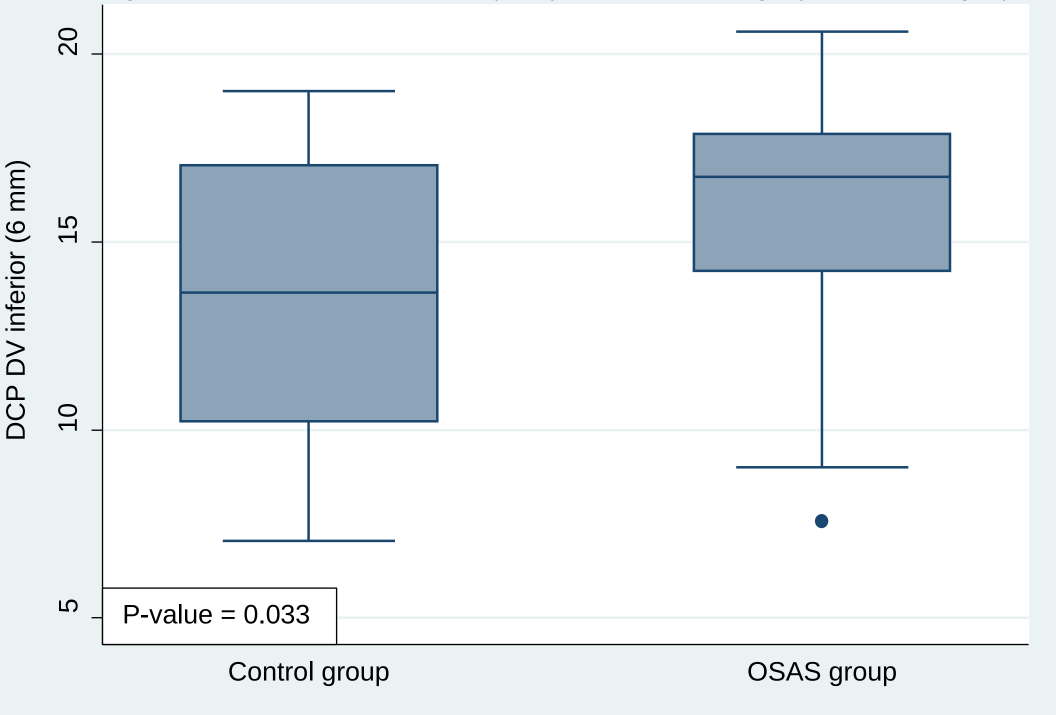

Figure 4: Differences in DCP DV parafoveolar ring between the control group and the OSAS group

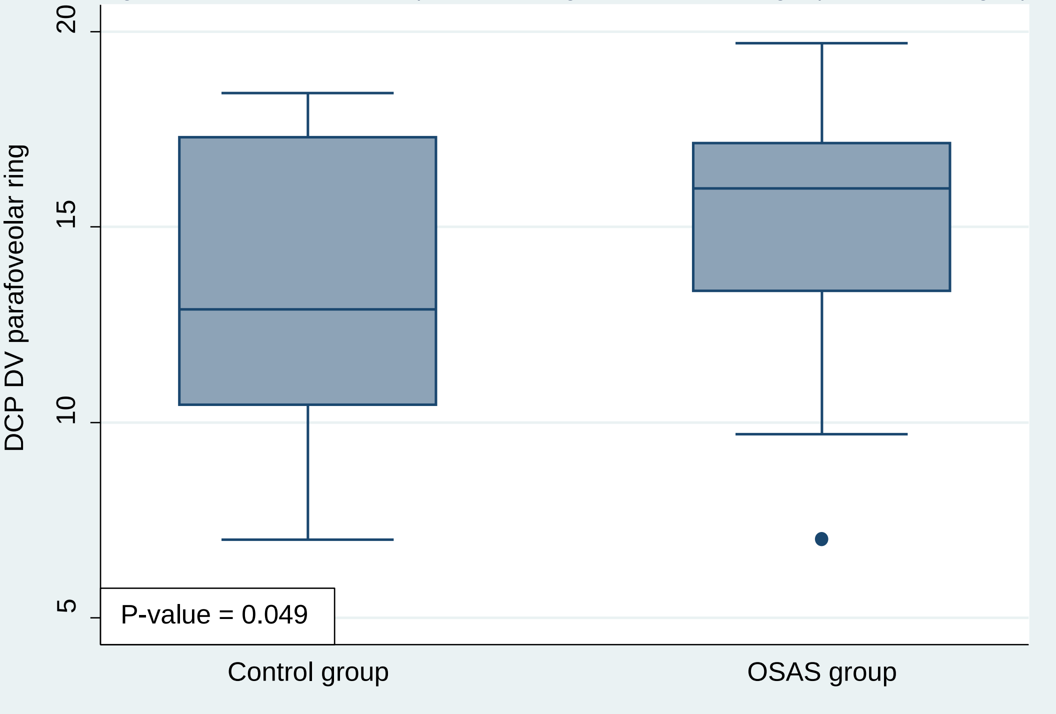

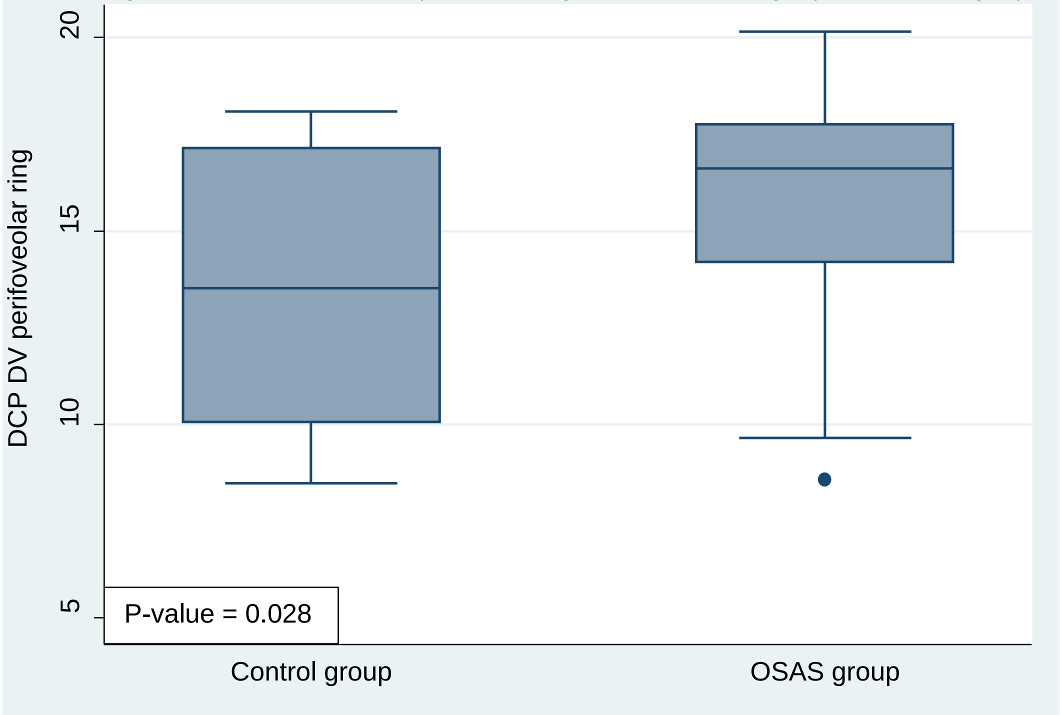

Figure 5: Differences in DCP DV perifoveolar ring between the control group and the OSAS group

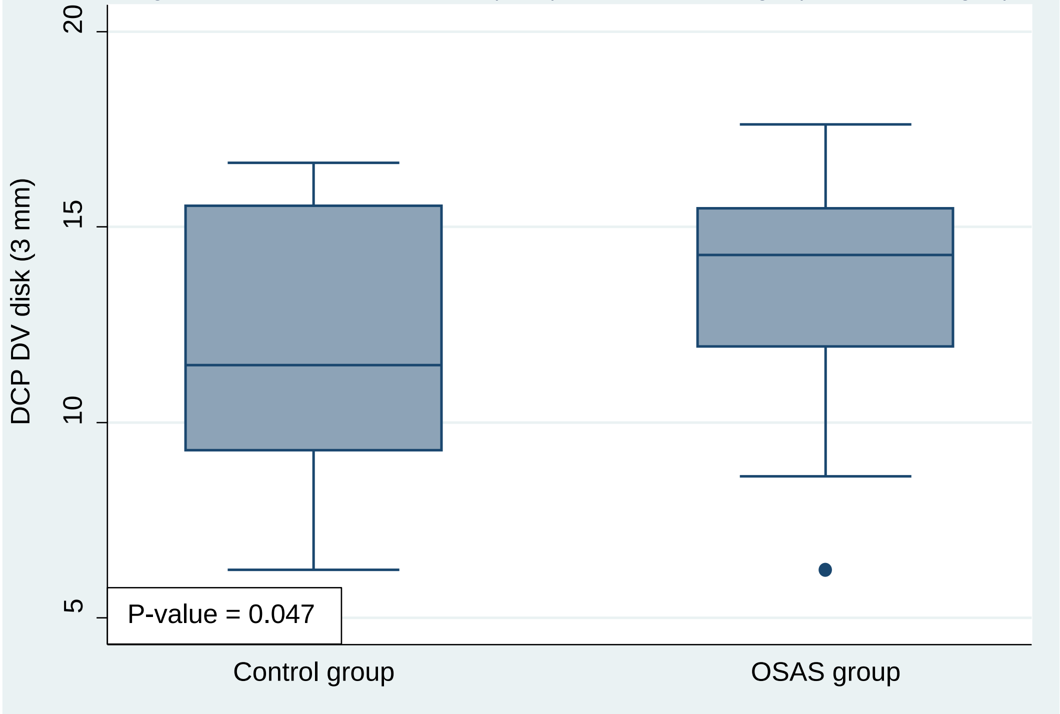

Figure 6: Differences in DCP DV disk (3 mm) between the control group and the OSAS group

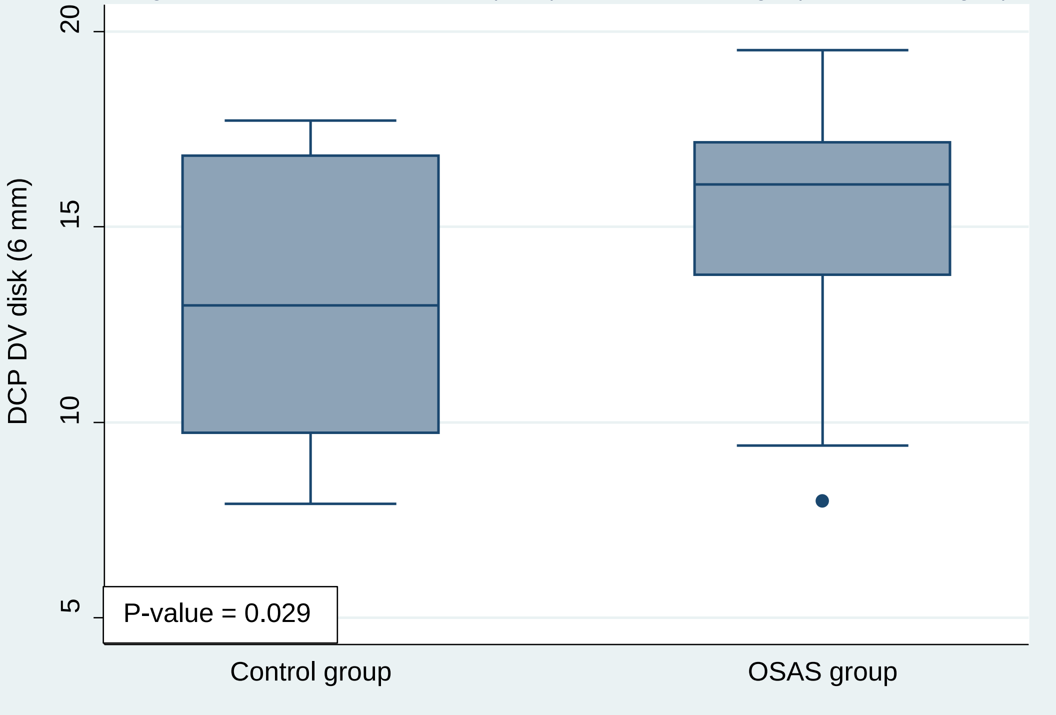

Figure 7: Differences in DCP DV disk (6 mm) between the control group and the OSAS group

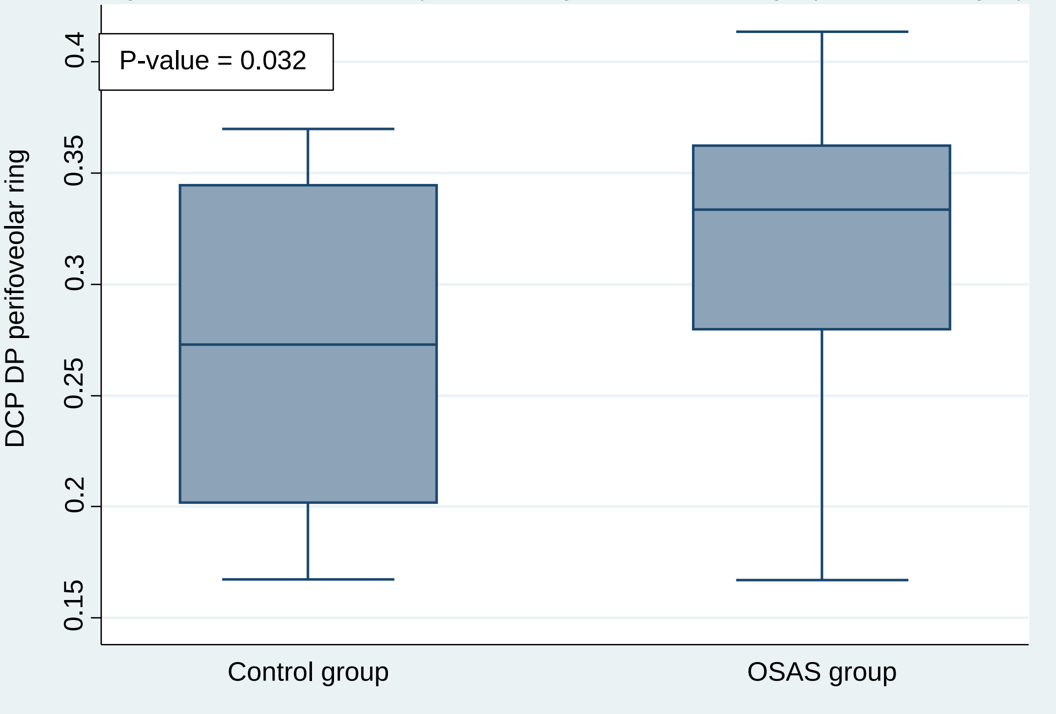

Figure 8: Differences in DCP DP perifoveolar ring between the control group and the OSAS group
